# Model of a Queuing Approach for Patient Accrual in Phase 1 Oncology Studies

**DOI:** 10.1001/jamanetworkopen.2020.4787

**Published:** 2020-05-13

**Authors:** Paul H. Frankel, Vincent Chung, Joseph Tuscano, Tanya Siddiqi, Sagus Sampath, Jeffrey Longmate, Susan Groshen, Edward M. Newman

**Affiliations:** 1 Division of Biostatistics, Department of Research Information Sciences, City of Hope, Duarte, California; 2 Department of Medical Oncology, City of Hope, Duarte, California; 3 Hematology/Oncology, University of California, Davis, Davis; 4 Hematology and Hematopoietic Cell Transplantation, City of Hope, Duarte, California; 5 Radiation Oncology, City of Hope, Duarte, California; 6 Biostatistics Core, Norris Cancer Center, University of Southern California, Los Angeles; 7 Developmental Cancer Therapeutics Program, Division of Molecular Pharmacology, City of Hope, Duarte, California

## Abstract

**Importance:**

Phase 1 cancer studies, which guide dose selection for subsequent studies, are almost 3 times more prevalent than phase 3 studies and have a median study duration considerably longer than 2 years, which constitutes a major component of drug development time.

**Objective:**

To discern a method to reduce the duration of phase 1 studies in adult and pediatric cancer studies without violating risk limits by better accommodating the accrual and evaluation process (or queue).

**Design:**

The process modeled, the phase 1 queue (IQ), includes patient interarrival time, screening, and dose-limiting toxicity evaluation. For this proof of principle, the rules of the 3 + 3 and rolling 6 phase 1 designs were modified to improve patient flow through the queue without exceeding the maximum risk permitted in the parent designs. The resulting designs, the IQ 3 + 3 and the IQ rolling 6, were each compared with their parent design by simulations in 12 different scenarios.

**Main Outcomes and Measures:**

(1) The time from study opening to determination of the maximum tolerated dose (MTD), (2) the number of patients treated to determine the MTD, and (3) the association of the design with the dose selected as the MTD.

**Results:**

Based on 800 simulations, for all 12 scenarios considered, the IQ 3 + 3 and the IQ rolling 6 designs were associated with reduced expected study durations compared with the parent design. The expected IQ 3 + 3 reduction ranged from 1.6 to 10.4 months (with 3.7 months for the standard scenario), and the expected reduction associated with IQ rolling 6 ranged from 0.4 to 10.5 months (with 3.4 months for the standard scenario). The increase in the mean number of patients treated in the IQ 3 + 3 compared with the 3 + 3 ranged from 0.6 to 3.2 patients. No increase in the number of patients was associated with the IQ rolling 6 compared with the rolling 6 design. The probability of selecting a dose level as the MTD changed by less than 3% for all dose levels and scenarios in both parent designs.

**Conclusions and Relevance:**

This study found that IQ designs were associated with reduced mean duration of phase 1 studies compared with their parent designs without changing the risk limits or MTD selection operating characteristics. These approaches have been successfully implemented in both hematology and solid tumor phase 1 studies.

## Key Points

**Question:**

Can the duration of phase 1 studies using either the 3 + 3 or rolling 6 designs be substantially reduced without exceeding the patient risk limits or changing the operating characteristics of the parent design?

**Findings:**

This decision analytical model found that the modified study designs were associated with reduced expected study durations. The modified designs were associated with minimal changes in the number of patients treated and the determination of the maximum tolerated dose, without changing the operating characteristics or exceeding the risk limits of the parent design.

**Meaning:**

Per this analysis, a substantial reduction in the time required to bring new advances to the clinic can be accomplished by simple modifications of 2 commonly used phase 1 trial designs.

## Teaser

This decision analytical model assesses a queuing approach to modify the design of phase 1 oncology trials.

## Introduction

While clinical trial timeliness factors into cost and relevance,^1,2^ phase 1 oncology trials are often underappreciated for their association with the time to translate a therapeutic advance into practice. Unlike most phase 1 studies, which take less than 1 year,^3^ phase 1 oncology studies take a median of approximately 32 months after study activation,^4^ and the number of phase 1 cancer clinical trials is consistently almost 3 times the number of phase 3 studies (eTable 1 in the Supplement). In addition, while multiple phase 2 and 3 studies can run in parallel, they must wait for the relevant phase 1 study to complete. Because phase 1 study duration is often slot limited and minimally affected by adding sites, we must look elsewhere for opportunities to alter this early bottleneck that delays therapeutic advances in oncology. Here, we focus on the patient queue and evaluation process to reduce phase 1 study duration. As a proof of principle, we focus on the traditional 3 + 3 phase 1 design^5^ and the rolling 6 design often used in pediatric studies.^6^

To our knowledge, this represents the most beneficial modification of 2 of the most commonly used designs (1 for adults and 1 for pediatric populations) to reduce expected study duration without affected the operating characteristics. These new designs have been successfully implemented in several completed and ongoing clinical trials.

## Methods

Phase 1 oncology designs typically consider only the first cycle of experimental treatment in both the determination of dose-limiting toxicities (DLTs) and the formal guidelines for dose-escalation decisions. To limit the number of patients at risk for a DLT, most phase 1 designs restrict the number of patients enrolled on the current dose level during the first cycle of experimental therapy. This is our starting point for the study of the phase 1 queue. The activities performed for the purposes of this simulation study are not considered to be human subjects research (per US Department of Health and Human Services regulation under the 45 CFR 46 Common Rule).

**Figure 1.**
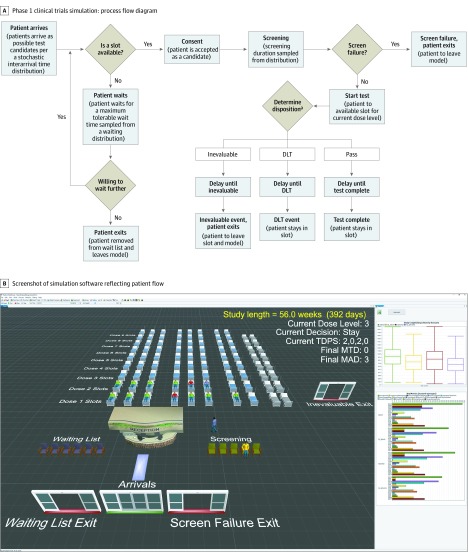
Representations of Patient Flow Through a Typical Phase 1 Trial A, The process accounts for 3 outcomes. Patients who receive protocol-specified minimum therapy without a dose-limiting toxicity (DLT) pass. Patients with a DLT are designated DLT. Inevaluable denotes ones who do not meet criteria for pass or DLT, often because of inadequate treatment or insufficient follow-up. Disposition is found by randomly sampling times of occurrence of inevaluable and DLT distributions. If both occur, the disposition is the first event. B, Simulation software showing patient flow. Each dose level has 2 cohorts of 3 beds, plus 2 extra beds for use in queue-based modifications during escalation. There are 2 more beds (gray) for rare instances during deescalation of the rolling 6 design and queue-based modified designs, so all consenting patients are treated without delay. Patients go to the waitlist or screening (yellow). If they exceed maximum wait time or do not meet criteria, they exit. Patients meeting criteria take a bed, are treated, and are pending (blue). Patients who pass are green; with DLTs, red. If inevaluable, they exit. A summary data dashboard for many of the same scenario and various design parameters is at right (https://www.flexsim.com/clinical-trials). MAD indicates maximum administered dose; MTD, maximum tolerated dose; TDPS, total, DLTs, pending, status (dose above closed).

Figure 1 illustrates the major steps involved in a phase 1 trial that form the basis for the patient queue that are reflected in the simulation tool. After each patient provides consent or any change occurs in any patient’s evaluation status, the protocol team decides the dose level and availability of slots for the accrual of additional patients based on the accumulated data and the protocol-specified phase 1 design. The decision can be to escalate (accrue at the next higher dose level), accrue at the same dose level, deescalate (accrue at the next lower dose level), hold accrual, or end accrual and either declare a maximum tolerated dose (MTD) or declare that the lowest dose level tested is too toxic. The phase 1 design guides these decisions.

We modified the 3 + 3 design and rolling 6 design decisions to better accommodate the phase 1 queue. The modifications were evaluated on the time and number of patients required to determine the MTD while constrained to (1) inherit the maximum level of patient risk associated with the parent design, (2) provide at least an equally rigorous assessment of toxicity, (3) maintain the parent design’s operating characteristics with respect to the MTD determination, and (4) revert to the parent design if accrual is slow or the principal investigator chooses to delay securing consent from patients when the queue-based designs permit accrual but the parent design does not.

The respective queue-based modifications (Table 1 and Table 2)^7,8^ are denoted the IQ 3 + 3 and the IQ rolling 6 designs. At any time, on the current dose level, there is the total number of patients enrolled (eg, promised or taking a slot, excluding those deemed inevaluable for DLT determination), the number of DLTs, and the number of patients evaluable. The number evaluable represents patients who were fully assessed and either had a DLT or did not (termed a *pass*), whereas the difference between total and evaluable numbers represents patients whose evaluations are pending. The column for the 3 + 3 design can been seen as representing the traditional rules: accrue 3 patients, escalate with 0 DLTs, deescalate with 2 DLTs, and expand to 6 patients with 1 DLT (in which 2 or more DLTs results in dose deescalation and 1 DLT in 6 patients results in dose escalation if the next higher dose is open, and the MTD requires 6 patients treated with 1 DLT at most). Both IQ-based modifications can have up to 8 patients per dose level during the escalation phase. In addition, when deescalating, applying the rule that all patients (up to as many as 10 patients, which occurs in <0.1% of simulations) should not be denied treatment once given a consent form are possible in both IQ designs.

**Table 1.** Comparison of the 3 + 3 and the Phase 1 Queue 3 + 3 Design Decisions^a^

Row	No. on current level			Dose level for next patient	
	Total^b^	Evaluable^c^	Dose-limiting Toxicity^d^	IQ 3 + 3^**e**^	3 + 3^**e**^
1	0-2	0	0	Same dose level	Same dose level
2	3	0	0	Hold accrual	Hold accrual
3	1-2	1	0	Same dose level	Same dose level
4	3	1	0	Same dose level	Hold accrual
5	4	1	0	Hold accrual	Not allowed
6	2	2	0	Same dose level	Same dose level
7	3	2	0	Same dose level	Hold accrual
8	4-5	2	0	Same dose level	Not allowed
9	6	2	0	Hold accrual	Not allowed
10	3	3	0	Escalate^f^	Escalate^f^
11	4-6	3-5	0	Escalate^f^	Same (up to 6; implies that higher dose cannot be tested further)
12	6	6	0	Escalate (or MTD)^f^	MTD
13	1-2	1	1	Same dose level	Same dose level
14	3	1	1	Hold accrual	Hold accrual
15	2	2	1	Same dose level	Same dose level
16	3	2	1	Same dose level	Hold accrual
17	4	2	1	Hold accrual	Not allowed
18	3-5	3-5	1	Same dose level	Same dose level
19	6	3	1	Hold accrual	Hold accrual
20	6	4	1	Same dose level	Hold accrual
21	6	5	1	Same dose level	Hold accrual
22	7	4	1	Hold accrual	Not allowed
23	7	5	1	Same dose level	Not allowed
24	6-8	6-8	1	Escalate^f^	Escalate (patients 7 and 8 not allowed)^f^
25	2-7	2-6	2	Deescalate^g^	Deescalate (patient 7 not allowed)^g^
26	7	7	2	MTD	Not allowed
27	8	7	2	Hold accrual	Not allowed
28	8	8	2	MTD	Not allowed
29	Any	Any	3	Deescalate^g^	Deescalate^g^

Abbreviations: IQ, phase 1 queue; MTD, maximum tolerated dose.

^a^ The IQ design and the corresponding parent design are side by side. Rare scenarios are not listed, but the full decision grid is available in the software input module available.^7,8^

^b^ The number of patients who consented to that dose level, excluding individuals who did not meet screening criteria or patients considered inevaluable with respect to dose-limiting toxicity.

^c^ The number of patients who provided an answer to the dose-limiting question (yes or no).

^d^ The number of patients who experienced a dose-limiting toxicity. The number pending is the difference between the total number and the number evaluable.

^e^ The action to be taken for the next patient for the IQ design and the parent design, respectively. If a patient pending evaluation on a lower dose experiences a dose-limiting toxicity, the principal investigator in consultation with the sponsor may choose to reduce the dose level of any patients currently on a higher dose level, pending review of the adverse event data.

^f^ If the next higher dose level is not available (there is no higher dose level or the higher dose level was already tested and found to be too toxic), a maximum of 8 patients can be treated at the current dose level and the principal investigator should declare the MTD with 0 or 1 dose-limiting toxicity of 6 (or 0 of 5) patients. For IQ 3 + 3, no more than 4 patients at risk are allowed, with no more than 6 patients at risk for the IQ rolling 6 design. Two dose-limiting toxicities in 7 or 8 patients means that the principal investigator can also declare the MTD (and it is suggested to continued using monitoring rules for the expanded cohort).

^g^ Current level exceeds the MTD. The MTD is the highest level at which less than 33% of patients had dose-limiting toxicities, with at least 6 patients evaluable.

**Table 2.** Comparison of Rolling 6 and Phase 1 Queue Rolling 6 Design Decisions^a^

Row	No. on current level			Dose level for next patient	
	Tota**l**^b^	Evaluaable^c^	Dose-limiting toxicities^d^	IQ rolling 6^e^	Rolling 6^e^
1	0-5	0	0	Same dose level	Same dose level
2	6	0	0	Hold accrual	Hold accrual
3	1-5	1	0	Same dose level	Same dose level
4	6	1	0	Same dose level	Hold accrual
5	7	1	0	Hold accrual	Not allowed
6	2-5	2	0	Same dose level	Same dose level
7	6-7	2	0	Same dose level	Hold accrual (patient 7 not allowed)
8	8	2	0	Hold accrual	Not allowed
9	3	3	0	Escalate^f^	Escalate
10	4-5	3	0	Escalate^f^	Same dose level
11	6	3	0	Escalate^f^	Hold accrual
12	7-8	3	0	Escalate^f^	Not allowed
13	4	4	0	Escalate^f^	Escalate
14	5	4	0	Escalate^f^	Same dose level
15	6-8	4	0	Escalate^f^	Hold accrual (patients 7 and 8 not allowed)
16	5	5	0	Escalate (or MTD)^f^	Escalate (or MTD)^f^
17	6	5	0	Escalate (or MTD)^f^	Escalate (or MTD)^f^
18	7-8	5	0	Escalate (or MTD)^f^	Not allowed
19	1-5	1-5	1	Same dose level	Same dose level
20	6	1-3	1	Hold accrual	Hold accrual
21	6	4	1	Same dose level	Hold accrual
22	6	5	1	Same dose level	Hold accrual
23	7	4	1	Hold accrual	Not allowed
24	7	5	1	Same dose level	Not allowed
25	6-8	6-8	1	Escalate^f^	Escalate (patients 7 and 8 not allowed)^f^
26	2-8	2-6	2	Deescalate^g^	Deescalate (patients 7 and 8 not allowed)^g^
27	7	7	2	MTD	Not allowed
28	8	7	2	Hold accrual	Not allowed
29	8	8	2	MTD	Not allowed
30	Any	any	3	Deescalate^g^	Deescalate^g^

Abbreviations: IQ, phase 1 queue; MTD, maximum tolerated dose.

^a^ The IQ design and the corresponding parent design are side by side. Rare scenarios are not listed, but the full decision grid is available in the software input module available.^7,8^

^b^ The number of patients who consented to that dose level, excluding individuals who did not meet screening criteria or patients considered inevaluable with respect to dose-limiting toxicity.

^c^ The number of patients who provided an answer to the dose-limiting question (yes or no).

^d^ The number of patients who experienced a dose-limiting toxicity. The number pending is the difference between the total number and the number evaluable.

^e^ The action to be taken for the next patient for the IQ design and the parent design, respectively. If a patient pending evaluation on a lower dose experiences a dose-limiting toxicity, the principal investigator in consultation with the sponsor may choose to reduce the dose level of any patients currently on a higher dose level, pending review of the adverse event data.

^f^ If the next higher dose level is not available (there is no higher dose level or the higher dose level was already tested and found to be too toxic), a maximum of 8 patients can be treated at the current dose level and the principal investigator should declare the MTD with 0 or 1 dose-limiting toxicity of 6 (or 0 of 5) patients. For IQ 3 + 3, no more than 4 patients at risk are allowed, with no more than 6 patients at risk for the IQ rolling 6 design. Two dose-limiting toxicities in 7 or 8 patients means that the principal investigator can also declare the MTD (and it is suggested to continued using monitoring rules for the expanded cohort).

^g^ Current level exceeds the MTD. The MTD is the highest level at which less than 33% of patients had dose-limiting toxicities, with at least 6 patients evaluable.

As an example modification, for the IQ 3 + 3 design (Table 1; row 4), enrolling a fourth patient at the same dose level, knowing that 1 of 3 patients has a pass with 2 patients pending, is considered less risky than putting 3 patients at risk without any patient data, as is permitted by the 3 + 3 design, so this modification does not exceed the risk allowed by the 3 + 3 but rather starts a patient through the process in case a patient who started earlier is considered not to meet screening criteria or becomes inevaluable. This is the key principle behind the IQ 3 + 3 design. Additional details and rationale for special IQ 3 + 3 decisions can be found in the eAppendix in the Supplement, including why and when up to 8 patients can be accrued during dose-level escalation.

For the IQ rolling 6 design (Table 2), the parent design allows 6 patients to be put at risk but does not allow escalation of the first 3 patients pass when there are patients pending. As a result, the rolling 6 does not achieve its anticipated speed advantage.^9^ The IQ rolling 6 allows escalation with 3 or more patients who are treated and fully evaluated with no DLTs, with the additional knowledge that none of the patients whose data are pending or have begun treatment have reported a DLT, which is consistent with the rules for the IQ 3 + 3 design with patients pending. The decision rules have also been modified so the IQ rolling 6 design improves patient flow but does not exceed the maximum risk permitted in the rolling 6 design (eAppendix in the Supplement).

Using decision tables 1 and 2, the operating characteristics of the 3 + 3 and IQ 3 + 3 designs and the rolling 6 and IQ rolling 6 designs were evaluated for 12 scenarios detailed in Table 3, each motivated by our clinical trial experience. In each scenario, we specify a starting dose level, the lowest and highest dose levels, and other parameters (Table 3). The maximum waiting time was assumed to be 0 in the simulations, representing patients who will not wait if slots are unavailable and instead will be accrued to a different phase 1 study (or treated outside of a study).

**Table 3.** Scenarios Simulated for the 3 + 3, Phase 1 Queue 3 + 3, Rolling 6, and Phase 1 Queue Rolling 6^a^

Characteristic	Scenario											
	A1: Standard phase 1	A2: Low inevaluability, phase 1	A3: High inevaluability, phase 1	A4: Standard ∆screen failure	A5: Standard ∆arrival	A6: Standard ∆CL	A7: Standard ∆toxicity	B: 21-d safety lead-in	C1: Second cycle DLT	C2: Second cycle DLT ∆arrival	C3: Second cycle DLT ∆in evaluability	D: IP phase 1
Starting dose level	2	2	2	2	2	2	2	2	3	3	3	1
Lowest possible dose level	1	1	1	1	1	1	1	1	1	1	1	1
Highest possible dose level	5	5	5	5	5	5	5	2	4	4	4	6
Course length, d	28	28	28	28	28	21	28	21	56	56	56	28
Maximum waiting list time, d	0	0	0	0	0	0	0	0	0	0	0	0
Screening duration, d	β(0,28,1,1)	β(0,28,1,1)	β(0,28,1,1)	β(0,28,1,1)	β(0,28,1,1)	β(0,28,1,1)	β(0,28,1,1)	β(0,28,1,1)	β(0,28,1,1)	β(0,28,1,1)	β(0,28,1,1)	β(0,90,1,1.97)
Screen failure probability, %	30	30	30	60	30	30	30	30	30	30	30	40
Probability of patient being inevaluable , %	20	3.6	44	20	20	20	20	20	66	66	33	7.5
Time until patient becomes inevaluable, d	β(0,CL,1,1)	β(0,CL,1,1)	β(0,CL,1,1)	β(0,CL,1,1)	β(0,CL,1,1)	β(0,CL,1,1)	β(0,CL,1,1)	β(0,CL,1,1)	β(0,CL,1,1)	β(0,CL,1,1)	β(0,CL,1,1)	β(0,CL,1,1)
Dose-limiting toxicity probability function	F(0.2, 8.5)	F(0.2, 8.5)	F(0.2, 8.5)	F(0.2, 8.5)	F(0.2,8.5)	F(0.2, 8.5)	F(0.2, 5.5)	F(0.2, 5.5)	F(0.2, 9.5)	F(0.2, 9.5)	F(0.2, 9.5)	F(0.2, 10.5)
Time until dose-limiting toxicity event, d	β(0,CL,1.5,1)	β(0,CL,1.5,1)	β(0,CL,1.5,1)	β(0,CL,1.5,1)	β(0,CL,1.5,1)	β(0,CL,1.5,1)	β(0,CL,1.5,1)	β(0,CL,1.5,1)	β(29,CL,1.5,1)	β(29,CL,1.5,1)	β(29,CL,1.5,1)	β(0,CL,1.5,1)
Patient interarrival time, d	exp(0,10,1)	exp(0,10,1)	exp(0,10,1)	exp(0,10,1)	exp(0,15,1)	exp (0,10,1)	exp (0,10,1)	exp(0,10,1)	exp(0,10,1)	exp (0,15,1)	exp (0,10,1)	exp(0,15,1)

Abbreviations: β, beta distribution; CL, course length; DLTs, dose-limiting toxicities; exp, exponential distribution with the second parameter as the mean; IP, intraperitoneal.

^a^ F(x,y) = 100 × (0.5 + atan(x × π × [current dose level − y])/π).

Scenarios A1 through A3 (Table 3) are based on our experience with phase 1 studies in the California Cancer Consortium, and scenarios A4 through A7 explore the results of changes in the screen failure rate, the interarrival time distribution, the course length, and the dose-toxicity association. For scenarios A1 through A3, we collected data on 14 phase 1 (or dose-finding portions of phase 2) cancer studies that had completed their phase 1 portions between October 2004 and November 2014 in the California Cancer Consortium. Along with the more traditional 3 + 3 designs, these studies include our published phase 1 study that first used a queue-based method^10^ and a study using isotonic regression-guided decisions in patients with organ dysfunction. One key parameter, largely independent of the design, that affected the duration of trials is the rate of inevaluability (the frequency with which patients who are treated cannot be assessed for DLTs and subsequent dose-level escalation decisions). In Skolnik et al,^6^ the median inevaluability rate for patients in pediatric phase 1 studies in the Children’s Oncology Group was 13.6% (range, 3.4%-22.7%). Our consortium experience with adult solid tumors suggests a higher rate of patients who are inevaluable; in the 14 completed studies reviewed, there were 375 patients with 75 patients inevaluable, representing a 20% overall rate (scenario A1), with considerable variability between studies (3.6% to 44.0% in scenarios A2 and A3, respectively). Scenario A1 (Table 3), our standard example, has 5 dose levels, has a 28-day DLT period (cycle 1), starts at level 2, identifies candidates for the study at a mean of every 10 days, has a 1-month maximum screening period (with a screen failure of 30%), and a 20% inevaluable rate.

Reasons for inevaluability in our experience included patient choice; non-dose-associated complications, including rapid disease progression; noncompliance with the protocol; changes in insurance status; drug supply issues; reaction to a poisonous bug bite; and late discovery of disease that rendered the patient ineligible. For another important parameter, the screen failure rate, which is not automatically tracked in the consortium, we reviewed single-institution phase 1 clinical trials at the center City of Hope. The screen failure rate was fairly consistent across the solid tumor studies at approximately 30%, which represented all causes that would result in a patient not receiving treatment after providing consent. This screen failure rate was used for all scenarios other than A4, in which we looked to demonstrate the result of an increase in screen failures, and scenario D, in which we modeled a specific clinical trial with a known screen failure rate.

Scenario B was provided to compare the simulation of the IQ designs with its respective parent design for a safety lead-in study. Scenario C1 is based on a study of blinatumomab and lenalidomide (NCT02568553). This study’s experimental treatment was introduced on cycle 2, increasing the number of inevaluable patients (whose data could not be considered for a dose-escalation decision), while simultaneously allowing patients to hold a slot for an extended period. Early in the study, two-thirds of the patients were inevaluable, and combined with the cycle 2 evaluation, this provides the opportunity for a very substantial improvement while using queue-based methods. We amended the study to change from a 3 + 3 design to an IQ 3 + 3 design, which was subsequently approved by the Cancer Therapy Evaluation Program and the central institutional review board based on the simulation work. Scenarios C2 and C3 are variations that show the result of changes in accrual rate or inevaluability rate.

Scenario D is based on a study of intraperitoneal chemotherapy (NCT00825201), in which surgery preceding chemotherapy increased the time a patient can occupy a slot; the cycle length was 28 days, but surgery and recovery could delay chemotherapy up to 90 days (screening time). Forty percent of the patients were ineligible per screening criteria, but only 7.5% were inevaluable for DLTs. We estimated the screening distribution and the arrival distribution from the study data.

For each design (3 + 3, IQ 3 + 3, rolling 6, and IQ rolling 6) scenarios were simulated 800 times, representing different random samplings of patient arrivals, DLT events (yes or no) based on the dose-toxicity DLT probability curve, DLT time (if a DLT occurred), evaluable results and time, and screening results and time. The inclusion of 800 samplings provides high confidence in the results (eg, a standard error <1.8%) while providing adequate numbers to observe unusual events (an occurrence rate of 0.3% will be observed with a probability >90%). Each sampling used the same patients arriving at the same time across the designs, and if the nth patient had a DLT while being treated at level 1 in the rolling 6 design, this same patient treated at level 2 in the IQ rolling 6 design would have a DLT because it is a higher dose level. Calculations were carried out in FlexSim HC version 5.3 (FlexSim Healthcare). The module is freely available online.^7^ Results are presented in graphical and tabular forms using means, medians, and ranges to be consistent with the Consolidated Health Economic Evaluation Reporting Standards (CHEERS) checklist for transparency on issues associated with analytic methods (eg, skewness), study parameters (eg, values, ranges) and outcomes (eg, mean differences).

## Results

**Figure 2.**
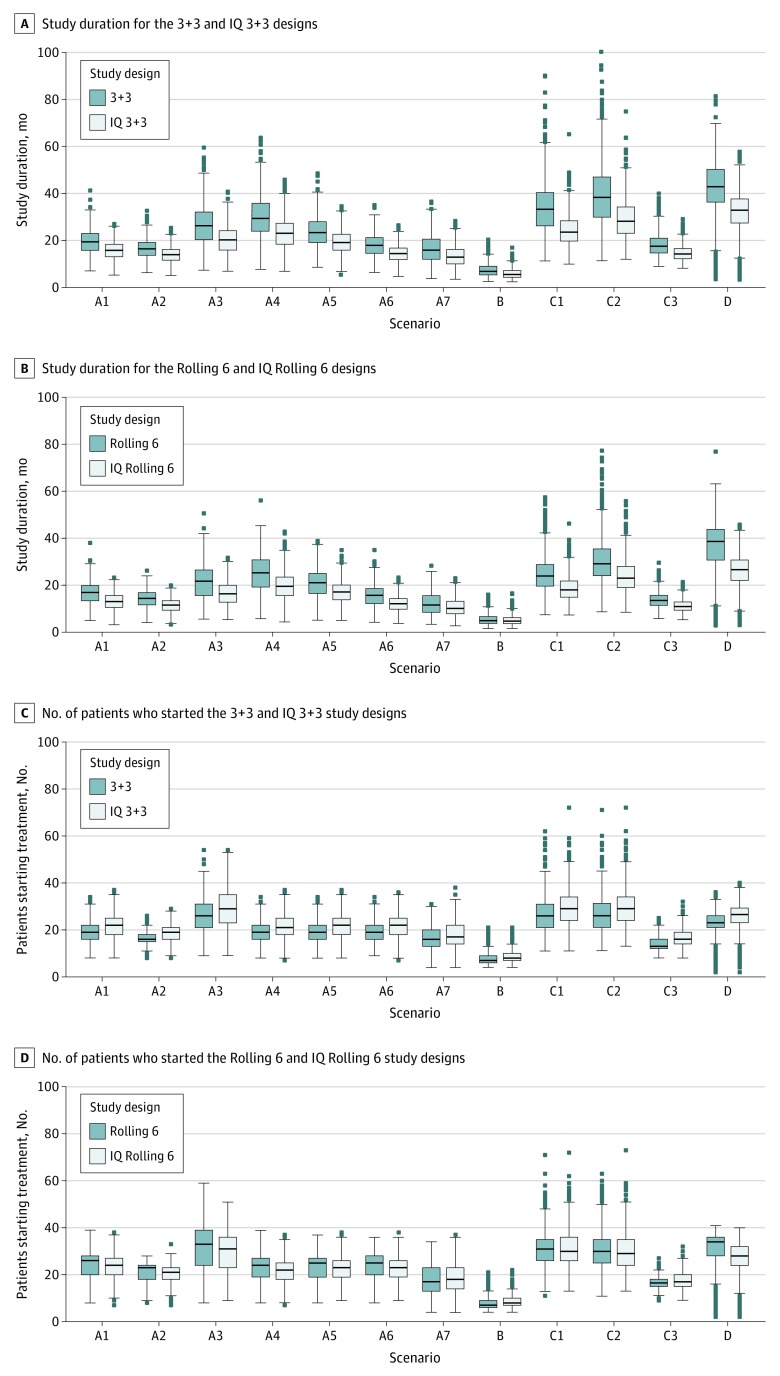
Box-Whisker Plots of Study Duration for the 3 + 3 and Phase 1 Queue (IQ) 3 + 3 and Rolling 6 and IQ Rolling 6 Designs A, Study duration for the 3 + 3 and IQ 3 + 3 studies. B, Study duration for the rolling 6 and IQ rolling 6 studies. C, Number of patients who started treatment for the 3 + 3 and IQ 3 + 3 designs. D, Number of patients who started treatment for the rolling 6 and IQ rolling 6 designs. Graphs are based on 800 simulations per scenario (Table 3).

For all scenarios, the IQ 3 + 3 design had shorter expected study durations than the 3 + 3 design, ranging from a reduction of 1.6 to 10.4 months (Figure 2A), and likewise, the IQ rolling 6 design has lower expected study durations than the rolling 6 design, ranging from a reduction of 0.4 months to 10.5 months (Figure 2B). There was a small increase in the mean number of patients treated on the IQ 3 + 3 design (difference in mean number of patients in all scenarios, <3.2 patients; Figure 2C), while the IQ rolling 6 had a smaller mean number of patients than the rolling 6 in 9 of the 12 scenarios, with a difference that did not exceed 3.3 patients (Figure 2D). Detailed results can be found in eTables 3 and 4 in the Supplement.

For scenario A1, the typical phase 1 study in the consortium, the expected (mean) duration of the phase 1 study using the traditional 3 + 3 design was 19.5 months (range, 7.1-41.3 months), whereas the expected duration was 15.8 months (range, 5.3-27.0 months) for the IQ 3 + 3 design. This represents an expected reduction of 3.7 months (difference in medians, 3.6 months; a 19% reduction). There is a mean increase of 2.8 patients. In eTable 5 in the Supplement, we extended this mean scenario to 9 dose levels, so the MTD would almost assuredly be reached before the highest allowable dose level. In that setting, the expected duration was reduced by 5.4 months (26.0 vs 20.6 months; a 21% reduction), and we also present additional metrics (expected percentage of patients on each dose level) that confirm that the operating characteristics of the 3 + 3 design are maintained.

By reducing inevaluability to 3.6% (scenario A2), the expected duration of both the 3 + 3 and the IQ 3 + 3 designs were shorter compared with scenario A1. The expected times were 16.5 vs 13.9 months, respectively (a 16% difference).

When increasing the rate of patients who are inevaluable to 44% (scenario A3), the expected duration of the 3 + 3 and the IQ 3 + 3 designs increased. The reduction because of the IQ-based design became 6.4 months (24%); compared with a reduction of 3.7 months (19%) associated with scenario A1.

With all designs, a higher number of patients who are inevaluable tended to reduce the dose level selected as the MTD (eTable 3 and 4 in the Supplement). This is because of the competing events of a patient experiencing a DLT and a patient being deemed inevaluable. As an extreme example, if the DLT evaluation period was very long, all patients would eventually be inevaluable, so the only patients who are evaluable would be those with a DLT before being deemed inevaluable, making all doses appear toxic.

In scenarios A4 through A7, we explored the results of (1) increasing screen failures, (2) increasing the availability of patients (reducing the interarrival time), (3) reducing the course length, and (4) increasing toxicity. The IQ designs had uniformly shorter expected durations (reduction of 6.7 months, 4.2 months, 3.7 months, and 3.1 months, respectively). The frequency of the selection of the MTD between the IQ design and the parent design differed by a range of less than 0.1% to 3%, and the mean number of DLTs at a dose level greater than the MTD differed by 0.01 to 0.10 patients.

For scenario B, which was considered the safety lead-in, even though 77.8% and 77.1% of the simulations selected the starting dose as the MTD (for the 3 + 3 and the IQ 3 + 3, respectively), the expected study duration was reduced from 7.6 months to 6.0 months (a 21% reduction). The expected number of patients differed by 0.6 patients.

For scenario C1, the expected study duration was reduced from 34.2 months to 24.5 months, a 9.7-month (28%) reduction. This was expected given the inevaluable rate of 66% (with a 30% screen failure rate) and the amount of time a patient will be pending (up to 84 days).

The expected number of patients required increased by 3.2, and the frequency of the MTD selected differed by less than 1%. The results of changing the arrival patterns or inevaluability rate are reflected in C2 and C3.

The last scenario, D, demonstrated a lengthier expected study duration of 42.1 months using the 3 + 3 design. This was 31.9 months with the IQ 3 + 3 design. The selection of the MTD differed by 0.5% to 1.5%.

For comparisons of the rolling 6 and IQ rolling 6 designs on the same 12 scenarios, a similar pattern emerged. For scenario A1, for example, the reduction in expected study duration for the IQ rolling 6 design is 3.4 months (16.4 vs 13.0 months), with a difference in the proportion of simulations choosing any particular dose as the MTD ranging from 0.1% to 1.1%; the difference in median study duration was 3.9 months. There were no extra patients treated, and in fact, the expected number of patients was reduced by 0.6 patients, reflecting that the rolling 6 design generally requires all patients treated to complete their assessment before escalating (even if 0 DLTs are noted in the first 3 patients), which adds time at that dose level and potentially results in even more patients at that dose level. For extended scenario A1 (eTable 5 in the Supplement), the expected reduction was 4.7 months.

## Discussion

The phase 1 queue depends on the treatment, patient population, and specific language in the protocol. For example, the screen failure rate is dependent on specific eligibility criteria, insurance issues, and the frailty of the patient population. Inevaluability for the purpose of DLT determination depends in part on the length of the evaluation period, the frailty of the patient population, the frequency of rapid disease progression, and other causes of drug discontinuation or unplanned dose reductions that are not associated with adverse events attributed to the dose of the investigational agent (or agents). Patients may also be considered inevaluable for DLT determination if critical tests are missed.

We observed in our phase 1 studies that these details can dramatically affect phase 1 study duration and such imperfections are present in every study. We can adapt designs to these imperfections to reduce the study duration while not exceeding the risk permitted in the parent design or affecting the operating characteristics. Because the rolling 6 design was originally developed for special settings, such as pediatrics, after the adult study, we compared the 3 + 3 with the IQ 3 + 3 design and the rolling 6 with the IQ rolling 6 design. In our typical phase 1 study, the IQ 3 + 3 design is expected to save 3.7 months in study duration compared with the 3 + 3 design, and the IQ rolling 6 design 3.4 months compared with the rolling 6 design, allowing phase 2 studies to start months earlier. Two scenarios (C1 or D) demonstrate approximate 10-month reductions in study duration when using the IQ 3 + 3 design.

The IQ modifications allow accrual of patients in certain situations when the parent design would not permit this, and in the case of the rolling 6 design, it permits escalation when the parent design would not because of patients who are pending, a key limitation of the original rolling 6 design rules. Several time-saving advantages include (1) the ability to more readily replace patients who do not meet screening criteria or become inevaluable for dose-escalation decisions; (2) if dose deescalation is required, the possibility that additional patients may have already been treated on the lower dose levels, accelerating the deescalation portion of the study; (3) the IQ rolling 6 design, which permits escalation with 0 of 3 or 0 of 4 patient with a DLT in the presence of additional patients who have started treatment without any DLT to date. Other advantages include situations in which physicians can treat a patient on the highest priority trial rather than hold accrual; especially for the IQ 3 + 3, a further advantage is to provide more information per dose level (if additional patients are treated prior to escalating), which can help select the recommended phase 2 dose. There is minimal difference between the parent and IQ designs in the selection of the MTD. Also, the principal investigator and/or treating physicians are not required to offer a trial to a patient if they wish to wait until the current patients at the current dose level complete cycle 1 evaluation. In that case, the IQ design reverts to the parent design.

eTable 2 in the Supplement lists several studies either with completed phase 1 portions (6 studies) or ongoing phase 1 portions (3 studies) using the IQ designs. This includes 2 lymphoma phase 1 trials (including the trial that motivated scenario C1); 2 hematology phase 1 studies, including a phase 1 stem cell transplant study^11^; 4 breast cancer studies; and an IQ rolling 6 study of escalating doses of radiation therapy to the prostate fossa,^12^ in which prior data were judged sufficient to allow 6 patients to accrue, analogous to the pediatric setting. After the first queue-based variation of the 3 + 3 design started accrual in May 2012,^10^ the experience of investigators, coordinating centers, and statistical teams with these designs have increased. Their use has increased, and additional refinements have been implemented that are reflected in the rules and simulations presented here.

The use of queuing methods to evaluate and optimize the operating characteristics of phase 1 designs or other aspects of clinical research is a nascent field. As with the parent designs, the determination of the MTD with the IQ variations is not usually the end of the dose-finding study. An expansion cohort at the recommended phase 2 dose or MTD is recommended with additional monitoring rules (as noted in the eAppendix in the Supplement for 1 study [NCT02568553]). When appropriate, a randomized dose-ranging phase 2 study evaluating candidate doses may also be suggested.^13^ Expanding the queue-based methods beyond the MTD determination is one possible area of future work. Queue-based modifications of DLT-rate targeting phase 1 designs with an implicit or explicit queue can also be explored.^14,15^ The direct cost of the IQ 3 + 3 design when compared with the traditional 3 + 3 design comes in the form of a few extra patients. There is no similar cost when converting the rolling 6 to the IQ rolling 6 design. However, the IQ rolling 6 design does allows escalation with patients pending, which is less conservative than the rolling 6 designs. Indirect costs for the IQ designs include more frequent review of the data and more clinical judgment as to whether to add new patients or escalate with patients pending (when permitted) or to hold accrual and revert to the parent design.

### Limitations

We did not model time to acquire data or decision time, which is in control of the data coordinating center and is usually short relative to the other intervals. We also did not implement separate screening-time distributions for patients considered screen failures vs successes or model delays in treatment.

## Conclusions

The IQ 3 + 3 and the IQ rolling 6 designs should be considered as alternatives to the 3 + 3 and rolling 6 designs, respectively. The IQ designs better adapt to the patient queue to reduce study duration without exceeding the risk limits of the parent design or affecting operating characteristics.

## Notes

Supplement. **eTable 1.** Phase I, II and III studies by year. **eTable 2.** Phase I or Safety Lead-in Clinical Trials Designed With Queue-based Methods. **eTable 3.** Simulations for 3+3 and IQ 3+3 (each based on 800 simulations). **eTable 4.** Simulations for Rolling 6 and IQ Rolling 6 (each based on 800 simulations). **eTable 5.** Simulations Based on Scenario A1 with 4 added higher dose levels (based on 800 simulations). **eAppendix.** Design Rationale.
