# Supplementary material for: Model of a Queuing Approach for Patient Accrual in Phase 1 Oncology Studies
Source: JAMA Netw Open. 2020 May 13;3(5):e204787. doi: 10.1001/jamanetworkopen.2020.4787 (PMC7221509; doi:10.1001/jamanetworkopen.2020.4787)
Supplement: Supplement. — eTable 1. Phase I, II and III studies by year. eTable 2. Phase I or Safety Lead-in Clinical Trials Designed With Queue-based Methods. eTable 3. Simulations for 3+3 and IQ 3+3 (each based on 800 simulations). eTable 4. Simulations for Rolling 6 and IQ Rolling 6 (each based on 800 simulations). eTable 5. Simulations Based on Scenario A1 with 4 added higher dose levels (based on 800 simulations). eAppendix. Design Rationale. [file jamanetwopen-3-e204787-s001.pdf]

Supplementary Online Content

Frankel PH, Chung V, Tuscano J, et al. Model of a queuing approach for patient accrual in phase I oncology studies. *JAMA Netw Open*. 2020;3(5):e204787. doi:10.1001/jamanetworkopen.2020.4787

eTable 1. Phase I, II and III studies by year.
eTable 2. Phase I or Safety Lead-in Clinical Trials Designed With Queue-based Methods.
eTable 3. Simulations for 3+3 and IQ 3+3 (each based on 800 simulations).
eTable 4. Simulations for Rolling 6 and IQ Rolling 6 (each based on 800 simulations).
eTable 5. Simulations Based on Scenario A1 with 4 added higher dose levels (based on 800 simulations).
eAppendix. Design Rationale.

This supplementary material has been provided by the authors to give readers additional information about their work.

© 2020 Frankel PH et al. *JAMA Network Open*.

eTable 1. Phase I, II and III studies by year:

Study Start	Phase I	Phase II*	Phase III
2019 (1/1/2019-10/11/2019)	1143	1552	395
2018	1278	1778	465
2017	1239	1751	429
2016	1208	1573	426
2015	1047	1499	482

*Phase II studies in clinicaltrials.gov can have a Phase I portion (e.g. safety lead-in), and can be Phase II/III studies, so we have focused on the number of pure Phase I studies (Early Phase 1 or Phase I) vs pure Phase III studies in clinicaltrials.gov (cancer, interventional, study start date).

eTable 2. Phase I or Safety Lead-in Clinical Trials Designed With Queue-based Methods

**NCT**	**Title**
NCT02568553	Lenalidomide and Blinatumomab in Treating Patients With Relapsed Non-Hodgkin Lymphoma
NCT00576979	Intensity-Modulated Radiation Therapy, Etoposide, and Cyclophosphamide Followed By Donor Stem Cell Transplant in Treating Patients With Relapsed or Refractory Acute Lymphoblastic Leukemia or Acute Myeloid Leukemia
NCT01567709	Alisertib in Combination With Vorinostat in Treating Patients With Relapsed or Recurrent Hodgkin Lymphoma, B-Cell Non-Hodgkin Lymphoma, or Peripheral T-Cell Lymphoma
NCT01041443	5-Fluoro-2'-Deoxycytidine and Tetrahydrouridine in Treating Patients With Acute Myeloid Leukemia or Myelodysplastic Syndromes
NCT02778685	Pembrolizumab, Letrozole, and Palbociclib in Treating Postmenopausal Patients With Newly Diagnosed Metastatic Stage IV Estrogen Receptor Positive Breast Cancer
NCT02648477	Pembrolizumab and Doxorubicin Hydrochloride or Anti-Estrogen Therapy in Treating Patients With Triple-Negative or Hormone Receptor-Positive Metastatic Breast Cancer
NCT02971761	Pembrolizumab and Enobosarm in Treating Patients With Androgen Receptor Positive Metastatic Triple Negative Breast Cancer

© 2020 Frankel PH et al. *JAMA Network Open*.

NCT01923506	Stereotactic Body Radiation Therapy in Treating Patients With Prostate Cancer After Undergoing Surgery
NCT03853707	Ipatasertib and Carboplatin With or Without Paclitaxel in Treating Patients With Metastatic Triple Negative Breast Cancer

eTable 3. Simulations for 3+3 and IQ 3+3 (each based on 800 simulations).

Variation	#Treated 3+3 Mean ,Med (range)	#Treated IQ3+3 Mean,Med (range)	#Months 3+3 Mean,Med (range)	#Months IQ3+3 Mean, Med (range)	Mean #DLTs above MTD	3+3 % MTD At level	IQ 3+3 % MTD At level	DLT rate (based on model)
**Scenario A1**<br>Ave Ineval<br>Phase I<br>20% Ineval	19.1, 19.0<br>(8-34)	21.9, 22.0<br>(8-37)	19.5, 19.4<br>(7.1-41.3)	15.8, 15.8<br>(5.3-27.0)	3+3: 0.87<br>IQ3: 0.95	NA <1.0%<br>1 7.4%<br>2 8.4%<br>3 9.9%<br>4 16.3%<br>5 58.0%	NA <1.0%<br>1 7.3%<br>2 9.3%<br>3 12.5%<br>4 15.6%<br>5 55.0%	NA NA<br>1 6.7%<br>2 7.6%<br>3 9.0%<br>4 10.8%<br>5 13.6%
**Scenario A2**<br>Low Ineval<br>Phase I<br>3.6% Ineval	16.1,16.0<br>(8-26)	18.6,19.0<br>(8-29)	16.5,16.5<br>(6.3-32.7)	13.9,14.0<br>(5.1-25.3)	3+3: 0.81<br>IQ3: 0.87	NA <1.0%<br>1 5.6%<br>2 7.8%<br>3 9.5%<br>4 15.4%<br>5 61.3%	NA <1.0%<br>1 6.1%<br>2 8.9%<br>3 10.0%<br>4 15.9%<br>5 58.5%	NA NA<br>1 6.7%<br>2 7.6%<br>3 9.0%<br>4 10.8%<br>5 13.6%
**Scenario A3**<br>High Ineval<br>Phase I<br>44% Ineval	26.0, 26.0<br>(9-54)	29.0, 29.0<br>(9-54)	26.6, 26.3<br>(7.3-59.6)	20.2, 20.3<br>(6.9-40.8)	3+3: 1.08<br>IQ3: 1.13	NA 1.0%<br>1 7.6%<br>2 11.8%<br>3 13.5%<br>4 17.6%<br>5 48.5%	NA 1.0%<br>1 8.4%<br>2 11.9%<br>3 15.0%<br>4 17.4%<br>5 46.4%	NA NA<br>1 6.7%<br>2 7.6%<br>3 9.0%<br>4 10.8%<br>5 13.6%
**Scenario A4**<br>Ave Ineval<br>Phase I<br>Δscreenfail to 60%	18.8, 19.0<br>(8-34)	21.1,21.0<br>(7-37)	30.0,29.4<br>(7.7-63.8)	23.3,23.1<br>(6.9-45.9)	3+3: 0.90<br>IQ3: 0.89	NA <1.0%<br>1 6.3%<br>2 8.3%<br>3 11.1%<br>4 17.1%<br>5 56.6%	NA <1.0%<br>1 6.6%<br>2 8.1%<br>3 12.1%<br>4 15.8%<br>5 56.9%	NA NA<br>1 6.7%<br>2 7.6%<br>3 9.0%<br>4 10.8%<br>5 13.6%
**Scenario A5**<br>Ave Ineval<br>Phase I<br>Δarrival (10 to 15 day mean)	19.0, 19.0<br>(8-34)	21.3, 22.0<br>(8-37)	23.5, 23.4<br>(8.6-48.5)	19.3, 19.1<br>(5.4-34.6)	3+3: 0.87<br>IQ3: 0.92	NA <1.0%<br>1 7.1%<br>2 8.5%<br>3 10.0%<br>4 16.3%<br>5 57.9%	NA <1.0%<br>1 6.8%<br>2 9.4%<br>3 12.6%<br>4 14.6%<br>5 56.1%	NA NA<br>1 6.7%<br>2 7.6%<br>3 9.0%<br>4 10.8%<br>5 13.6%
**Scenario A6**<br>Ave Ineval<br>Phase I<br>ΔCL to 21 days	19.0,19.0<br>(9-34)	21.4,22.0<br>(7-36)	18.1, 17.9<br>(6.4-35.1)	14.4, 14.4<br>(4.7-26.4)	3+3: 0.88<br>IQ3: 0.93	NA <1.0%<br>1 7.3%<br>2 8.4%<br>3 9.8%<br>4 16.5%<br>5 57.8%	NA <1.0%<br>1 7.1%<br>2 8.6%<br>3 12.3%<br>4 15.6%<br>5 55.9%	NA NA<br>1 6.7%<br>2 7.6%<br>3 9.0%<br>4 10.8%<br>5 13.6%
**Scenario A7**<br>Ave Ineval<br>Phase I<br>ΔToxicity	16.6, 16.0<br>(4-31)	18.1, 17.0<br>(4-38)	16.5, 15.9<br>(3.8-36.5)	13.4, 12.9<br>(3.5-28.3)	3+3: 2.1<br>IQ3: 2.2	NA 3.8%<br>1 15.4%<br>2 23.5%<br>3 27.8%<br>4 22.5%<br>5 7.1%	NA 4.4%<br>1 16.0%<br>2 24.3%<br>3 29.8%<br>4 20.6%<br>5 5.0%	NA NA<br>1 10.8%<br>2 13.6%<br>3 18.0%<br>4 25.9%<br>5 40.3%
**Scenario B**<br>Safety Lead-in Phase I	8.2, 7.0<br>(4-21)	8.8, 8.0<br>(4-21)	7.6, 6.9<br>(2.5-20.4)	6.0, 5.5<br>(2.4-17.0)	3+3: 0.53<br>IQ3: 0.56	NA 4.3%<br>1 18.0%<br>2 77.8%	NA 4.6%<br>1 18.3%<br>2 77.1%	NA NA<br>1 10.8%<br>2 13.6%
**Scenario C1**<br>2nd Cycle with experimental combination	26.7, 26.0<br>(11-62)	29.9, 29.0<br>(11-72)	34.2, 33.3<br>(11.2-90.2)	24.5, 23.6<br>(9.9-65.3)	3+3: 0.48<br>IQ3: 0.51	NA <1%<br>1 <1%<br>2 7.8%<br>3 13%<br>4 78.1%	NA <1%<br>1 1.3%<br>2 8.1%<br>3 13%<br>4 77.4%	NA NA<br>1 5.9%<br>2 6.7%<br>3 7.6%<br>4 9.0%
**Scenario C2**<br>2nd Cycle<br><br>Δarrival to 15	26.7, 26.0<br>(11-71)	29.6, 29.0<br>(13-72)	39.5, 38.4<br>(11.4-100.4)	29.1, 28.2<br>(12.0-75.0)	3+3: 0.47<br>IQ3: 0.48	NA <1%<br>1 1%<br>2 6.5%<br>3 12.3%	NA <1%<br>1 <1%<br>2 8.1%<br>3 13.3%	NA NA<br>1 5.9%<br>2 6.7%<br>3 7.6%

© 2020 Frankel PH et al. *JAMA Network Open*.

						4 79.0%	4 77.8%	4 9.0%
**Scenario C3**<br>2nd Cycle<br><br>Δineval rate to 33%	13.9, 13.0<br>(8-25)	16.5, 16.0<br>(8-32)	18.2, 17.6<br>(9.0-40.0)	14.6, 14.3<br>(8.2-29.1)	3+3: 0.32<br>IQ3: 0.36	NA <1.0%<br>1 <1.0%<br>2 6.5%<br>3 9.0%<br>4 84.4%	NA <1.0%<br>1 <1.0%<br>2 6.4%<br>3 10.6%<br>4 82.6%	NA NA<br>1 5.9%<br>2 6.7%<br>3 7.6%<br>4 9.0%
**Scenario D**<br>IP Phase I	22.5, 23.0<br>(2-36)	25.3, 26.5<br>(2-40)	42.1, 42.9<br>(3.5-81.5)	31.9, 32.9<br>(3.5-57.8)	3+3:.71<br>IQ3:.75	NA 3.6%<br>1 3.5%<br>2 4.6%<br>3 5.9%<br>4 6.5%<br>5 10.9%<br>6 65.0%	NA 3.8%<br>1 3.6%<br>2 4.1%<br>3 7.4%<br>4 6.6%<br>5 10.9%<br>6 63.6%	NA NA<br>1 5.3%<br>2 5.9%<br>3 6.7%<br>4 7.6%<br>5 9.0%<br>6 10.8%

eTable 4. Simulations for Rolling 6 and IQ Rolling 6 (each based on 800 simulations).

Variation	#Treated Rolling 6 Mean,Med (range)	#Treated IQ R6 Mean,Med (range)	#Months Rolling 6 Mean,Med (range)	#Months IQ R6 Mean, Med (range)	Mean #DLTs above MTD	Rolling 6 % MTD At level	IQ R6 % MTD At level	DLT rate (based on model)
**Scenario A1**<br>Ave Ineval<br>Phase I<br>20% Ineval	23.9, 26.0<br>(8-39)	23.3, 24.0<br>(7-38)	16.4,16.9<br>(4.9-38.0)	13.0,13.0<br>(3.1-23.2)	R6 : 0.89<br>IQR: 0.94	NA <1.0%<br>1 7.6%<br>2 9.0%<br>3 12%<br>4 14.9%<br>5 56.0%	NA <1.0%<br>1 7.8%<br>2 8.9%<br>3 13%<br>4 15.4%<br>5 54.9%	NA NA<br>1 6.7%<br>2 7.6%<br>3 9.0%<br>4 10.8%<br>5 13.6%
**Scenario A2**<br>Low Ineval<br>Phase I<br>3.6% Ineval	20.5,23.0<br>(8-28)	20.1,21.0<br>(7-33)	14.0,14.4<br>(4.1-26.2)	11.4,11.6<br>(3.2-19.8)	R6 : 0.90<br>IQR: 0.88	NA <1.0%<br>1 6.5%<br>2 9.3%<br>3 13.1%<br>4 14.9%<br>5 55.8%	NA <1.0%<br>1 6.5%<br>2 9.3%<br>3 11.9%<br>4 14.0%<br>5 57.8%	NA NA<br>1 6.7%<br>2 7.6%<br>3 9.0%<br>4 10.8%<br>5 13.6%
**Scenario A3**<br>High Ineval<br>Phase I<br>44% Ineval	31.5,33.0<br>(8-59)	30.0,31.0<br>(9-51)	21.4,21.7<br>(5.6-50.6)	16.4,16.3<br>(5.3-31.6)	R6 : 1.06<br>IQR:1.13	NA 1.0%<br>1 8.1%<br>2 14.3%<br>3 14.0%<br>4 14.3%<br>5 48.4%	NA 1.1%<br>1 7.9%<br>2 12.3%<br>3 15.5%<br>4 17.0%<br>5 46.3%	NA NA<br>1 6.7%<br>2 7.6%<br>3 9.0%<br>4 10.8%<br>5 13.6%
**Scenario A4**<br>Ave Ineval<br>Phase I<br>Δscreenfail to 60%	23.1,24.0<br>(8-39)	21.8,22.0<br>(7-37)	25.2,25.3<br>(5.7-56.1)	19.8,19.5<br>(4.3-42.7)	R6 : 0.93<br>IQR:0.94	NA <1.0%<br>1 7.5%<br>2 8.1%<br>3 13.8%<br>4 15.4%<br>5 54.5%	NA < 1.0%<br>1 7.1%<br>2 8.6%<br>3 12.9%<br>4 15.8%<br>5 55.3%	NA NA<br>1 6.7%<br>2 7.6%<br>3 9.0%<br>4 10.8%<br>5 13.6%
**Scenario A5**<br>Ave Ineval<br>Phase I<br>Δarrival (10 to 15 day mean)	23.2,25.0<br>(8-37)	22.1,23.0<br>(9-38)	20.7,21.0<br>(5.2-38.8)	17.1,17.1<br>(4.9-34.9)	R6 : 0.91<br>IQR:0.94	NA < 1.0%<br>1 7.3%<br>2 8.6%<br>3 12.8%<br>4 15.4%<br>5 55.5%	NA < 1.0%<br>1 6.9%<br>2 9.4%<br>3 12.6%<br>4 15.1%<br>5 55.4%	NA NA<br>1 6.7%<br>2 7.6%<br>3 9.0%<br>4 10.8%<br>5 13.6%
**Scenario A6**<br>Ave Ineval<br>Phase I<br>ΔCL to 21 days	23.5,25.0<br>(8-36)	22.5,23.0<br>(9-38)	15.4,15.7<br>(4.2-35.0)	12.1,12.1<br>(3.7-23.2)	R6 : 0.91<br>IQR: 0.91	NA < 1.0%<br>1 7.8%<br>2 8.4%<br>3 13.1%<br>4 14.6%<br>5 55.6%	NA < 1.0%<br>1 7.5%<br>2 8.5%<br>3 12.5%<br>4 14.5%<br>5 56.8%	NA NA<br>1 6.7%<br>2 7.6%<br>3 9.0%<br>4 10.8%<br>5 13.6%
**Scenario A7**<br>Ave Ineval<br>Phase I<br>ΔToxicity	18.0,17.0<br>(4-34)	18.7,18.0<br>(4-37)	12.3,11.6<br>(3.4-28.3)	10.7,10.1<br>(2.7-22.9)	R6 : 2.01<br>IQR:2.16	NA 3.5%<br>1 18.8%<br>2 26.1%<br>3 26.8%<br>4 19.5%<br>5 5.4%	NA 3.9%<br>1 17.4%<br>2 24.8%<br>3 28.3%<br>4 21.1%<br>5 4.6%	NA NA<br>1 10.8%<br>2 13.6%<br>3 18.0%<br>4 25.9%<br>5 40.3%
**Scenario B**<br>Safety Lead-in Phase I	8.3, 7.0<br>(4-21)	9.0, 8.0<br>(4-22)	5.5, 4.9<br>(1.6-15.8)	5.1, 4.7<br>(1.6-16.4)	R6 : 0.53<br>IQR: 0.57	NA 3.3%<br>1 18.9%<br>2 77.9%	NA 4.3%<br>1 18.9%<br>2 76.9%	NA NA<br>1 10.8%<br>2 13.6%
**Scenario C1**<br>2nd Cycle with experimental combination	31.1, 31.0<br>(11-71)	31.1, 30.0<br>(13-72)	24.9, 23.9<br>(7.3-57.4)	18.7, 18.0<br>(7.3-46.2)	R6 : 0.48<br>IQR: 0.47	NA <1.0%<br>1 <1.0%<br>2 9.6%<br>3 12.0%<br>4 77.5%	NA <1.0%<br>1 <1.0%<br>2 7.5%<br>3 12.8%<br>4 78.8%	NA NA<br>1 5.9%<br>2 6.7%<br>3 7.6%<br>4 9.0%

© 2020 Frankel PH et al. *JAMA Network Open*.

**Scenario C2**<br>2nd Cycle<br><br>Δarrival to 15	30.7, 30.0<br>(11-63)	30.0, 29.0<br>(13-73)	30.4, 29.1<br>(8.6-77.3)	23.9, 23.0<br>(8.5-55.8)	R6 : 0.49<br>IQR: 0.47	NA <1.0%<br>1 1.0%<br>2 8.8%<br>3 12.6%<br>4 77.4%	NA <1.0%<br>1 <1.0%<br>2 7.9%<br>3 12.6%<br>4 78.5%	NA NA<br>1 5.9%<br>2 6.7%<br>3 7.6%<br>4 9.0%
**Scenario C3**<br>2nd Cycle<br><br>Δineval rate to 33%	16.7, 16.5<br>(9-27)	17.7, 17.0<br>(9-32)	13.8, 13.5<br>(5.8-29.6)	11.2, 10.9<br>(5.2-21.1)	R6 : 0.36<br>IQR: 0.39	NA <1.0%<br>1 <1.0%<br>2 7.3%<br>3 9.8%<br>4 82.8%	NA <1.0%<br>1 <1.0%<br>2 7.0%<br>3 11.0%<br>4 81.6%	NA NA<br>1 5.9%<br>2 6.7%<br>3 7.6%<br>4 9.0%
**Scenario D**<br>IP Phase I	30.0, 34.0<br>(2-41)	26.7, 28.0<br>(2-40)	36.1, 38.6<br>(3.1-76.9)	25.6, 26.6<br>(3.1-45.7)	R6 : 0.78<br>IQR: 0.74	NA 4.4%<br>1 4.6%<br>2 6.4%<br>3 6.0%<br>4 7.1%<br>5 10.5%<br>6 61.0%	NA 4.0%<br>1 4.5%<br>2 4.4%<br>3 7.3%<br>4 6.3%<br>5 10.0%<br>6 63.6%	NA NA<br>1 5.3%<br>2 5.9%<br>3 6.7%<br>4 7.6%<br>5 9.0%<br>6 10.8%

eTable 5. Simulations Based on Scenario A1 with 4 added higher dose levels (based on 800 simulations).

Based on Model Scenario A1 with an additional 4 higher dose levels		#Treated<br>3+3<br>Mean=25.6<br>Median=26.0<br>Range 8-53<br><br>Average fraction at dose level:	#Treated<br>IQ3+3<br>Mean=28.4<br>Median=28.5<br>Range 8-58<br><br>Average Fraction at dose level:	#Treated<br>Rolling 6<br>Mean=31.6<br>Median=33.0<br>Range 8-62<br><br>Average Fraction at dose level:	#Treated<br>IQ R6<br>Mean=30.3<br>Median=31.0<br>Range 7-66<br><br>Average Fraction at dose level:	3+3<br>% MTD<br>At level	IQ 3+3<br>% MTD<br>At level	Rolling 6<br>% MTD<br>At level	IQ R6<br>% MTD<br>At level
Dose level	DLT Rate								
*NA	NA	--	--	--	--	<1.0%	<1.0%	<1.0%	<1.0%
1	6.7%	0.04	0.04	0.04	0.04	7.4%	7.3%	7.6%	7.8%
2	7.6%	0.22	0.24	0.26	0.24	8.4%	9.3%	9.0%	8.9%
3	9.0%	0.19	0.21	0.22	0.22	9.9%	12.4%	12.1%	13.0%
4	10.8%	0.17	0.17	0.17	0.17	14.3%	14.1%	14.8%	13.8%
5	13.6%	0.14	0.14	0.13	0.13	17.3%	18.6%	14.8%	17.8%
6	18.0%	0.11	0.11	0.09	0.10	21.1%	20.9%	22.0%	20.8%
7	25.9%	0.08	0.07	0.06	0.06	16.6%	14.6%	15.6%	14.0%
8	40.3%	0.04	0.02	0.02	0.02	4.7%	2.1%	3.4%	3.8%
9	59.7%	0.01	<0.01	<0.01	<0.01	<1%	<1%	<1%	<1%

*NA represents when dose level 1 was above the MTD

Study Duration (months)	3+3	IQ3+3	Rolling 6	IQ R6
Study Duration (Mean or Expected)	26.0	20.6	21.5	16.8
Study Duration Median (Range)	25.7<br>(7.1-63.9)	20.7<br>(5.3-42.6)	22.2<br>(4.9-45.9)	16.7<br>(3.1-36.4)
#Started Treatment<br>(Mean or Expected)	25.6	28.4	31.6	30.4
#Started Treatment<br>Median (Range)	26.0<br>(8-53)	28.5<br>(8-58)	33 .0<br>(8-62)	31.0<br>(7-66)
#DLTs above MTD<br>(Mean or Expected)	2.2	2.2	2.0	2.2
#DLTs above MTD<br>Median (range)	2.0<br>(0-7)	2.0<br>(0-6)	2.0<br>(0-5)	2.0<br>(0-7)

eAppendix. Design Rationale:

IQ 3+3 Rationale:

Row 4 differences were discussed in the manuscript. Additionally, When 2 patients have a "Pass" (fully assessed and evaluable with no DLT) a fifth patient can be accrued without

exceeding the 3+3 risks. In that situation the worst case scenario in the 3+3 is that third patient has a DLT, in which case, 3 additional patients can be accrued. As a result, with 2 patients without a DLT, we are within the risk limits to accrue up to 4 additional patients, allowing us to get to 6 enrolled, with 2 evaluable each with a "Pass" (rows 7-8). In row 11, with 4-6 treated and 3-5 evaluable with no DLT, the 3+3 would have previously escalated if 0/3, so the 3+3 could only be in that situation if expanding to 6 patients with the dose above closed. That is not automatically the case with the IQ 3+3, however, we would know that there were no DLTs in at least 3 patients, and no DLTs in the pending patients reported to date, so if the above level is open, we can escalate, with a stronger safety signal than with only information on 3 patients, hence reduced risk to future patients. The next major difference (row 16) occurs with three patients accrued (consented and promised a slot), 1 DLT and 1 patient with a "Pass" (or 1 of the 2 evaluable patients with a DLT). The 3+3 holds accrual in that setting, whereas the IQ 3+3 can accrue a 4th patient. In the standard 3+3, if 1 of 1 patients have a DLT, 2 additional patients can be accrued, so we are within that risk to have 2 patients accrued with 1 of 2 patients with a DLT, again, not exceeding the risks of the 3+3. Risk-based logic also allows the IQ 3+3 to accrue additional patients with 1 DLT, and 4 or 5 patients evaluable and up to 2 patients pending (rows 20, 21). The IQ 3+3 also allows escalation with 1 out of 6 evaluable patients with DLTs and no DLTs reported in pending patients.

To consider why eight patients are permitted on the IQ 3+3 consider: If we have 3 patients enrolled, 2 with a "PASS" (meaning fully evaluable with no DLT), and 1 DLT, we can enroll another 3 patients per the standard 3+3. If the 4th patient is a "PASS", we are safer with 1 of 4 DLT than 1 of 3, so we are not exceeding the risks to still enroll 3 patients. That means we could get to 7 patients. If the 5th patient is "PASS", the same argument can be made, and we get to 8 patients. However, if a 6th patient is a "PASS" we escalate, so we don't need more than 8 slots

during escalation.

Also note that if the patient consented and was promised a slot based on a dose level but has not started treatment when an escalation has been declared, that patient is put on the currently accruing (higher) dose level, and this is modelled in the simulations. To address DLTs occurring in pending patients at a lower dose level after escalation we note that information would not have been observed in the 3+3, so any conservative action reduces the risk to future patients when compared to the original 3+3.

IQ Rolling 6

Where the original Rolling 6 allows 6 patients to be put at risk at the same time, the IQ Rolling 6 maintains that risk limit or lower, and when 1 patient has a "Pass" or a second patient has a "Pass", an additional 1 or 2 patients, respectively, can be accrued to maintain 6 patients at risk when there are no DLTs. If 1 patient has a DLT, the Rolling 6 permits accrual of up to 5 additional patients, as does the IQ Rolling 6. If 1 patient has a DLT, the IQ Rolling 6 as presented is not maximally aggressive. When there is a DLT, we revert to the risk rules of the 3+3 for accruing beyond 6 patients due to the conditions for using the Rolling 6 being in question in the presence of a DLT[6].

Additional Details:

Note 1:

Some decisions in Table 1-2 required special notes. For example, the traditional 3+3 design and the Rolling 6 design do not specify what to do with 2 DLTs in 7 patients or 2 DLTs in 8 patients (row 26 and 28 in Table 1 and rows 27 and 29 in Table 2). We first addressed this situation in the context of the NCI-CTEP sponsored study (NCT02568553, Table S1). Considerations

© 2020 Frankel PH et al. *JAMA Network Open*.

included (1) the principal investigator did not want to be obligated to lower the dose if 2 of 7 patients had DLTs and wanted the option to continue to treat at that dose (but not escalate), suggesting that dose as the MTD; (2) additional principal investigators agreed; (3) as recommended[8], the study had additional monitoring rules for the expansion cohort so that if 3 or more DLTs occurred in the first 12 patients treated at that dose, the recommended Phase 2 dose and continued accrual would be re-evaluated; (4) Children's Oncology Group has set a precedent allowing the possibility of cohort expansion when 2 DLTs occur in 6 evaluable patients if the two DLTs are of different classes (e.g. hepatotoxicity and myelosuppression) with internal review (personal communication); and (5) we searched for logic consistent with the decisions inherent in the 3+3 design using two beta distributions for the probability of a DLT (analogous to an optimistic and pessimistic prior) and two decision thresholds for escalation or de-escalation. We challenged those sets of rules consistent with the 3+3 with 2 DLTs out of 7 patients. We found that if 1/6 was an acceptable DLT rate not requiring de-escalation, the decision would never be to de-escalate (see Note 3 below) resulting in 2 DLTs out of 7 or 8 patients constituting the MTD in our decision grid for the IQ 3+3 and the IQ Rolling 6.

Note 2:

We currently allow the protocol team to evaluate the specifics of the lower level DLT(s), and permit the team to immediately reduce the dose on the patients at the higher dose or allow patients on the higher dose level to complete their evaluation and then decide what action to take. The simulations do not consider such flexible decision-making and only considers those DLTs that occurred on a lower dose after escalating if later de-escalation re-visits that dose. The frequency of two DLTs at a dose below the MTD in the simulations in either the IQ 3+3 or the IQ Rolling 6 is rare (~2%).

© 2020 Frankel PH et al. *JAMA Network Open*.

Note 3:

R-CODE for 2 of 7 Question

```
comment<- c("
•            The Beta distribution is a flexible distribution for the probability of a DLT.
•    This will be used for the probability of a DLT (p) using parameters a, b, e.g. Beta(a,b)
•            The expected value of p, E(p) = a/(a+b).
•            We update the expected value of p with patient data on a dose according to:
•            E(p) = (a+ #DLTs)/ (a+#Pts+b), (e.g. if a=b=1 with no patients, E(p)=1/2) .
•            To generate a logical decision engine that agrees with 3+3 based on two priors:

A) One prior, beta(a1,b1) that guides whether the dose is safe and we can continue to treat.
Based on a threshold (T1) for the expected DLT rate
B) One prior, beta(a2,b2) guides whether we are comfortable escalating the dose.
Based on a different threshold (T2) for the expected DLT rate.

(a1+#DLTS)/(a1+#PTS+b1)>T1?    Go Down
(a2+#DLTS)/(a2+#PTS+b2)<T2?    Go Up
Otherwise stay


•            55,000,000 random samples of parameters to find two priors beta(a1,b1), beta(a2,b2),
and
    two threshold (T1,T2), that are consistent with the traditional 3+3 rules. We used a general
    grid search.

•            Find sets of parameters matched the 3+3
•            Apply all different sets of rules to 2 DLTs of 7 patients.
(a1+2)/(a1+7+b1)>T1?    Go Down ..else
(a2+2)/(a2+7+b2)<T2?    Go Up …else MTD

")

# Use 64-BIT R to allow larger number of simulations

set.seed(9)
simnum<-55000000  # 55 million


#(a1+2)/(a1+7+b1)>T1?    Go Down ..else
#(a2+2)/(a2+7+b2)<T2?    Go Up …else MTD
a1=runif(simnum,min=0.00,max=13)  # rep(0,simnum) #rbinom(simnum,10,0.2)
b1= runif(simnum,min=0.0,max=50)
a2=runif(simnum,min=0.0,max=45)
b2=runif(simnum,min=0,max=45)
t1=runif(simnum,min=0,max=1/2)  # 1/6 was key
t2= runif(simnum,min=0,max=1/2)  # 1/3
```

© 2020 Frankel PH et al. *JAMA Network Open*.

```
######
down<-function(ndlt,tot) {return( ((a1+ndlt)/(a1+tot+b1))>t1  ) }
up<-function(ndlt,tot) {return( ((a2+ndlt)/(a2+tot+b2))<t2 ) }

# Flawed with 0 DLTS
z0<-( down(0,0) | up(0,0)   | down(0,1) | up(0,1)   | down(0,2) | up(0,2)   | down(0,3)  | !up(0,3)  |
      down(0,4) | !up(0,4)  | down(0,5) |!up(0,5)  | down(0,6)| !up(0,6)   )

# Flawed with 1 DLT
z1<- ( down(1,1) | up(1,1) | down(1,2) | up(1,2) | down(1,3) | up(1,3) | down(1,4) | up(1,4) |
down(1,5) | up(1,5) |
      down(1,6) | !up(1,6) )

# Flawed with 2 DLTs
z2<-( !down(2,2) | up(2,2) | !down(2,3) | up(2,3) | !down(2,4) | up(2,4) | !down(2,5) | up(2,5) |
!down(2,6) | up(2,6) )

# Flawed with 3 DLTs
z3<- (!down(3,3) | up(3,3) | !down(3,4) | up(3,4) | !down(3,5) | up(3,5) | !down(3,6) | up(3,6) )

# Flawed with 4 DLTs
z4<- (!down(4,4) | up(4,4) | !down(4,5) | up(4,5) | !down(4,6) | up(4,6) )

# Flawed with either 0, 1, ,2 ,3 or 4 DLTs (not consistent with 3+3)
bad<- (z0 | z1 | z2 | z3 | z4)

# Gather up data
tot<-data.frame(bad, a1, a2, b1, b2, t1, t2, prior1=a1/(a1+b1),
         prior2=a2/(a2+b2))

# Select parameters that are consistent with the 3+3
tot2<-tot[!tot$bad,]
dim(tot2)[1]/simnum
# Of paramters consistent with 3+3,  answer is 1297 with above conditions and seed
dim(tot2)[1]

##################################################################################
#######



downcheck<-function(ndlt,tot,a1c,b1c,t1c) {return( ((a1c+ndlt)/(a1c+tot+b1c))>t1c ) }
upcheck<-function(ndlt,tot,a2c,b2c,t2c) {return( ((a2c+ndlt)/(a2c+tot+b2c))<t2c) }

# Ask what those 1297 sets of parameters would do with 2 DLTs out of 7 patients
tot3<-data.frame(tot2, d2of7=downcheck(2,7,tot2$a1,tot2$b1,tot2$t1),
                  u2of7=upcheck(2,7,tot2$a2,tot2$b2,tot2$t2) )
table(tot3$u2of7)
# 0/1297  go up
```

© 2020 Frankel PH et al. *JAMA Network Open*.

```
table(tot3$d2of7)
# 959/1297 go down  BUT
downtot<-tot3[tot3$d2of7,]
summary(downtot$t1) # All decisions to go down had the threshold to go down less than 1/6

# If 1/6 is an acceptable rate, the stay option is selected.
# Refining the grid to increase the number of parameter sets consistent with the 3+3 does not
# Alter the conclusion.
```

© 2020 Frankel PH et al. *JAMA Network Open*.
